# Objective assessment of nasal obstruction

**DOI:** 10.20407/fmj.2021-029

**Published:** 2022-07-22

**Authors:** Kensei Naito, Seiji Horibe, Yosuke Tanabe, Hisayuki Kato, Satoshi Yoshioka, Ichiro Tateya

**Affiliations:** 1 Fujita Academy, Toyoake, Aichi, Japan; 2 Miyanomori ENT Clinic, Nagoya, Aichi, Japan; 3 Department of Otolaryngology Head and Neck Surgery, School of Medicine, Fujita Health University, Toyoake, Aichi, Japan

**Keywords:** Nasal obstruction, Rhinomanometry, Acoustic rhinometry, Nasal allergy, Obstructive sleep apnea

## Abstract

There are many methods and types of equipment for measuring the nasal airway, but there is no consensus regarding the results of various clinical studies on nasal obstruction. In this review, we discuss the two major methods of objectively assessing the nasal airway: rhinomanometry and acoustic rhinometry. The Japanese standard of rhinomanometry in Japanese adults and children was established by the Japanese Standardization Committee on Rhinomanometry in 2001 and 2018, respectively. However, the International Standardization Committee has proposed different standards because of differences in race, equipment, and social health insurance systems. The standardization of acoustic rhinometry in Japanese adults is making progress in several Japanese institutes, but the international standardization of acoustic rhinometry has not yet begun. Rhinomanometry is the physiological expression of nasal airway breathing, whereas acoustic rhinometry is the anatomic expression. In this review, we introduce the history and methods of the objective assessment of nasal patency and the physiological and pathological issues regarding nasal obstruction.

## 1. Introduction

Nasal obstruction is a well-known manifestation of many nasal diseases, such as allergic, infectious, hypertrophic, and atrophic rhinitis; acute and chronic sinusitis; nasal polyps; nasal septal deviation and perforation; nasal and paranasal sinus tumors; stenotic malformations; and psychiatric nasal obstruction. Nasal obstruction induces mouth breathing, causing dryness of the mouth; dysosmia or anosmia with taste problems; nasal speech; headache; reduced attentiveness; snoring; daytime sleepiness; and obstructive sleep apnea (OSA). How do we objectively evaluate nasal obstruction? How do objective and subjective nasal congestion relate to each other? Are there any human racial differences in nasal patency?

The Department of Otolaryngology at Fujita Health University has conducted many investigations in an effort to answer these questions. As active members of the International Standardization Committee on Objective Assessment of the Nasal Airway (ISCOANA)^1–4^ and the Japanese Standardization Committee on Objective Assessment of the Nasal Airway (JSCOANA),^5,6^ we have been dedicated to the development of this field since each committee was established. In this review, we discuss many of our major achievements, including studies on the objective assessment of nasal obstruction.

## 2. History of objective assessment of nasal patency

**Figure 1:**
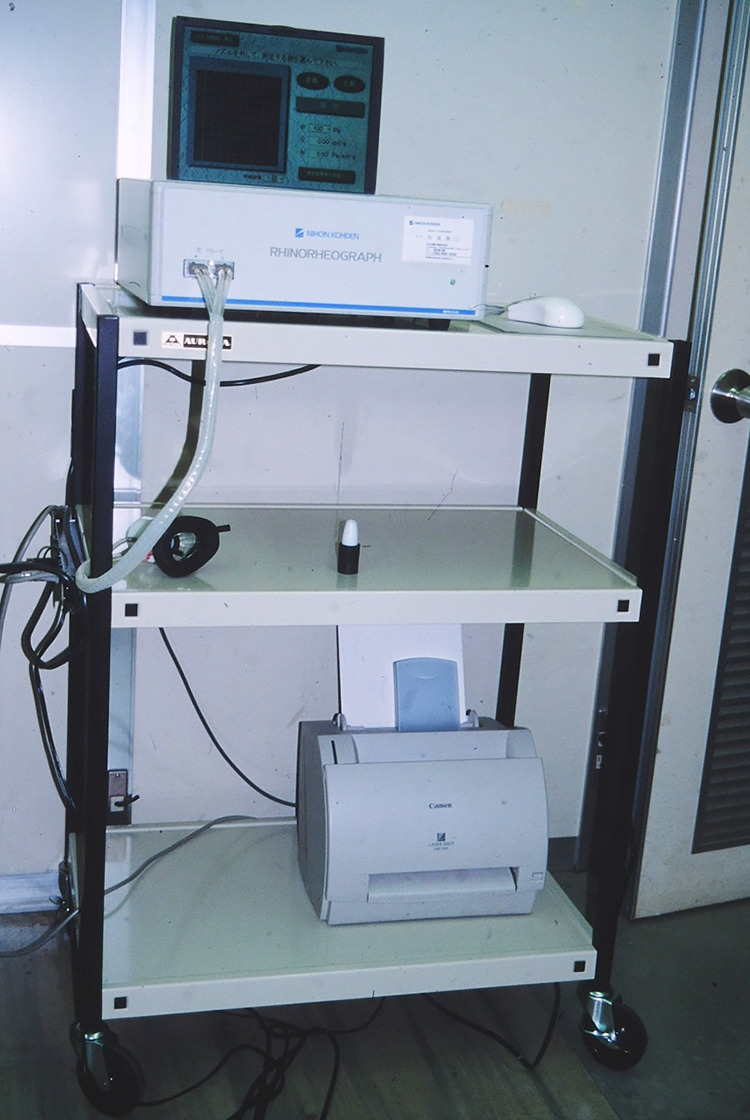
MPR-3100 rhinomanometer (Nihon Kohden Corporation, Tokyo, Japan).

In the early phase of the objective assessment of nasal patency, Zwaardemaker^7^ placed a cold mirror immediately under the nostril and observed the mirror for fogging by expiratory nasal airflow. In 1895, Kayser^8^ used the aerodynamic concept to assess nasal airflow. Rhinomanometry was developed as a superior method of objectively assessing nasal patency. Rhinomanometry is classified as either active or passive, and these two types are further divided into anterior and posterior techniques.^9^ The use of passive rhinomanometry has been gradually declining because of its unnaturalness and instability.^9,10^ Active rhinomanometry is a well-established method of objectively evaluating nasal patency (Fig. 1). Pneumotachographic systems are widely used to simultaneously measure nasal airflow ($\dot{V}$, cm^3^/s) and the differential transnasal pressure between the anterior nostril and the posterior choana (*ΔP*, Pa) of quiet nasal breathing. Nasal resistance (*R*, Pa/cm^3^/s) is calculated as an objective expression of nasal patency:

(1) $$R=\Delta P/\dot{V}\text{.}$$

Initially, active posterior rhinomanometry was a standard technique because of its logical measurability of instantaneous bilateral nasal resistance (*T*) (see Section 3.1). In recent decades, however, this method has been somewhat neglected because of its failure to measure postnasal pressure via the oral cavity.^10–12^ Active anterior rhinomanometry samples the postnasal pressure in one nasal cavity with the nostril occluded instead of the oropharynx. Several researchers have found this method to be easier than active posterior rhinomanometry for many clinicians and recommend it as a standard method.^2–4,10–14^

Nasal resistance (*R*), commonly calculated using Eq. 1, is applied to laminar flow. However, the actual alternating nasal airflow is considered nonlaminar, even under resting conditions.^15–17^ Despite the efforts of many investigators,^16,18,19^ this anomaly between empirical measurement and Eq. 1 remains subject to discussion.

Some researchers^18,20,21^ have measured *R* at a flow of 500 cm^3^/s, but many patients with nasal obstruction do not attain this flow level.^10,22^ Other groups^18,19^ have measured *R* at *ΔP*=150 Pa as the ideal point during spontaneous nasal breathing, but some regard this as relatively high for evaluation.^23,24^ Ohki and Hasegawa^25^ found that 100 Pa was more suitable for the Japanese adult population. *R* at 75 Pa must be much smaller than that calculated at former points.^26^ To avoid problems associated with predetermined pressure or airflow coordinates, we measured *R* using Eq. 1 at peak flow during quiet nasal breathing.^27^ Cole et al.^28^ computed *R* from averaged consecutive 50-Hz pressure and flow values (time-averaged method). The time-averaged *R* was almost identical to that obtained from Eq. 1 at peak flow, and *R* at 150 Pa was smaller than that obtained from the former two methods.^29,30^

Naito and Unno^31^ estimated the area under the pressure–flow curve (integrated *R*), and Broms et al.^32^ measured the angles at which the curves crossed circles at predetermined radii. Unfortunately, these two methods do not seem able to logically represent the true *R*.

**Figure 2:**
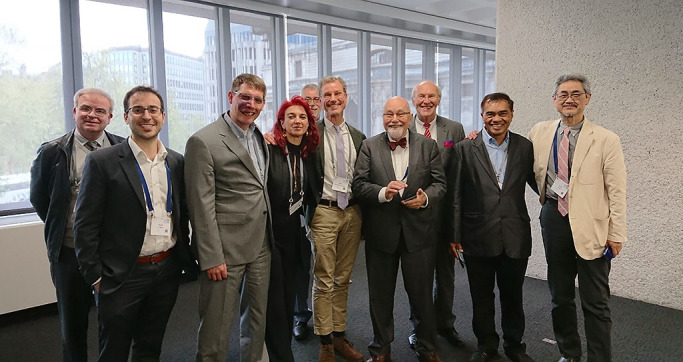
The latest International Standardization Committee on Objective Assessment of the Nasal Airway (ISCOANA) business meeting held at the Queen Elizabeth II Center, London, UK, on 25 April 2018.

**Figure 3:**
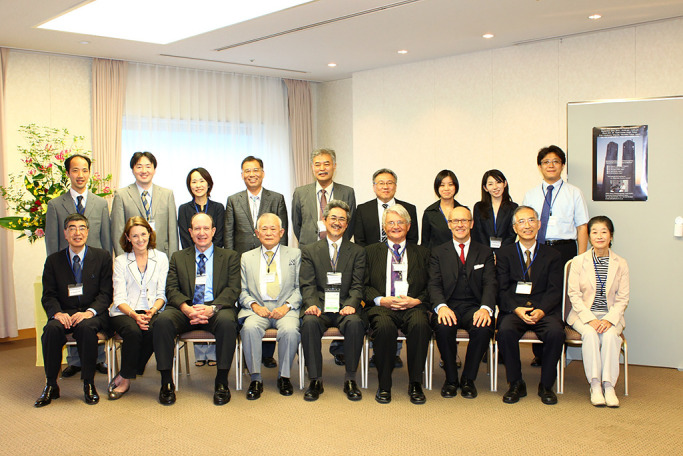
The first international meeting of objective assessment of the nasal airway by experts in this field in Tokyo, Japan, sponsored by the Department of Otolaryngology, School of Medicine, Fujita Health University, on 23 September 2011.

This variety of methods complicates investigations.^33^ Additionally, racial differences are an international problem.^34–36^ ISCOANA was established to recommend standardized methods of measuring and expressing rhinomanometric results (Fig. 2).^1–4^ In 1993, JSCOANA of the Japanese Rhinologic Society was established to focus Japanese opinions in the ISCOANA meeting.^5^ Naito, the first author of this review, attended international meetings from 2008 to 2019 as a representative of JSCOANA.^5^ In 2011, our department was a main sponsor of an international meeting on objective assessment of nasal airflow in Tokyo, Japan (Fig. 3).

In 1989, acoustic rhinometry was developed. This technique depicts consecutive sectional areas of the nasal cavity and intranasal volume measured by sound reflection from the equipment.^37^ Expression of the width of the nasal cavity as the objective nasal airway may be easier to understand, but this method also has several unresolved problems. Furthermore, the relationships between rhinomanometric results, which are physiological and dynamic expressions, and acoustic rhinometric evaluations, which are anatomical and static expressions, must be discussed. JSCOANA is currently investigating acoustic rhinometry–averaged results in normal Japanese adults. The relationship between these objective assessments (rhinomanometry and acoustic rhinometry) and patients’ perception of nasal congestion should also be discussed.

## 3. Fundamental and clinical investigations

### 3.1. Methods of rhinomanometry

Passive anterior and posterior rhinomanometry is not physiological because artificial air is forcibly compressed into the nose from the anterior nostril or, conversely, from the oropharynx to the nasal cavity, and the grades of the air forced through the nose are evaluated.^9,10^ Active rhinomanometry, in which *ΔP* and $\dot{V}$ are simultaneously measured during breathing through the nose, is more physiological. The main expression of the two parameters is *R* $\left(\Delta P/\dot{V}\right)$. Fundamentally and logically, many investigators believe that active posterior rhinomanometry is more suitable^11–13^; however, this method has been somewhat neglected because of failure to measure postnasal pressure via the oral cavity. It is much easier to measure *R* using active anterior rhinomanometry, which has been recommended as a standard method of rhinomanometry by ISCOANA and JSCOANA. Unfortunately, however, this method cannot measure *R* in patients with septal perforation and complete unilateral nasal obstruction.^38^

Although active posterior rhinomanometry can measure instantaneous bilateral nasal resistance (*T*), only unilateral *R* can be measured. *T* can be calculated using the following equation of Ohm’s law for parallel resistors:

(2) 1/T=1/R+1/LorT=R×L/(R+L),

where *R* is nasal resistance on the right side and *L* is nasal resistance on the left side.^39^

During active anterior rhinomanometry, there may be differences between the postnasal pressures acquired from the unmeasured nostril and the oropharynx as well as differences between *T*, *R*, and *L* calculated from Eq. 2 and the actual measurements. We compared the postnasal pressures in the nasopharynx with those in the anterior nostril and found no significant differences.^38^ In addition, we could fit *T* calculated from Eq. 2 with direct measurements:

With resistance at 100 Pa,

(3) $$T=1.07{\left[R/L/(R+L)\right]}^{0.77}\text{.}$$

With resistance by the time-averaged method,

(4) $$T=1.07{\left[R\times L/(R+L)\right]}^{0.92}\text{.}$$

We could then obtain the exact *T* measured by active anterior rhinomanometry instead of actual measurements by active posterior rhinomanometry.

As mentioned previously, active anterior rhinomanometry cannot be performed on patients with septal perforation or complete unilateral nasal obstruction.^38^ Instead, active posterior rhinomanometry is required in these patients. However, because this method sometimes fails to measure oropharyngeal pressure, we recommend applying a fine nasal catheter (8-French infant-feeding nasal catheter) through the nasal cavity to the nasopharynx instead of applying a mouth tube.^40^ This method can measure *R* much more easily than using a mouth tube, and the *R* values obtained from the mouth and nasal catheter are almost identical. However, although the catheter is fine, we still need to consider irritable stimulation by the catheter; this is especially true in patients with allergic rhinitis, who usually have hypersensitivity of the nasal mucosa.

**Figure 4:**
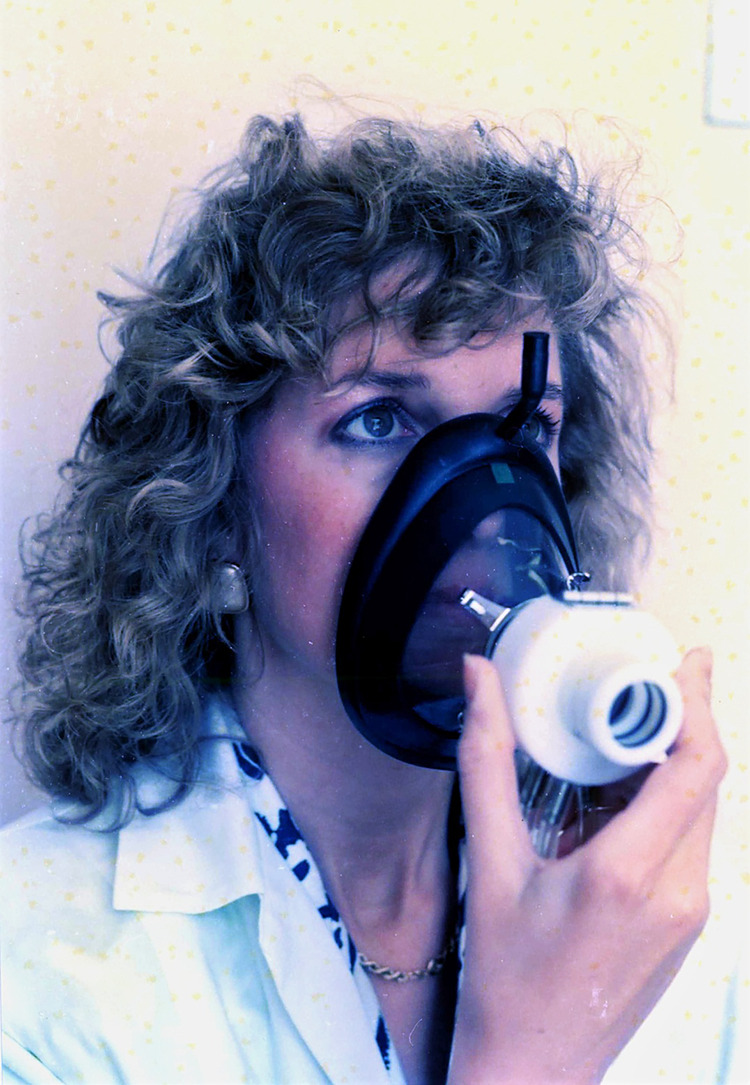
Active rhinomanometry with a mask.

**Figure 5:**
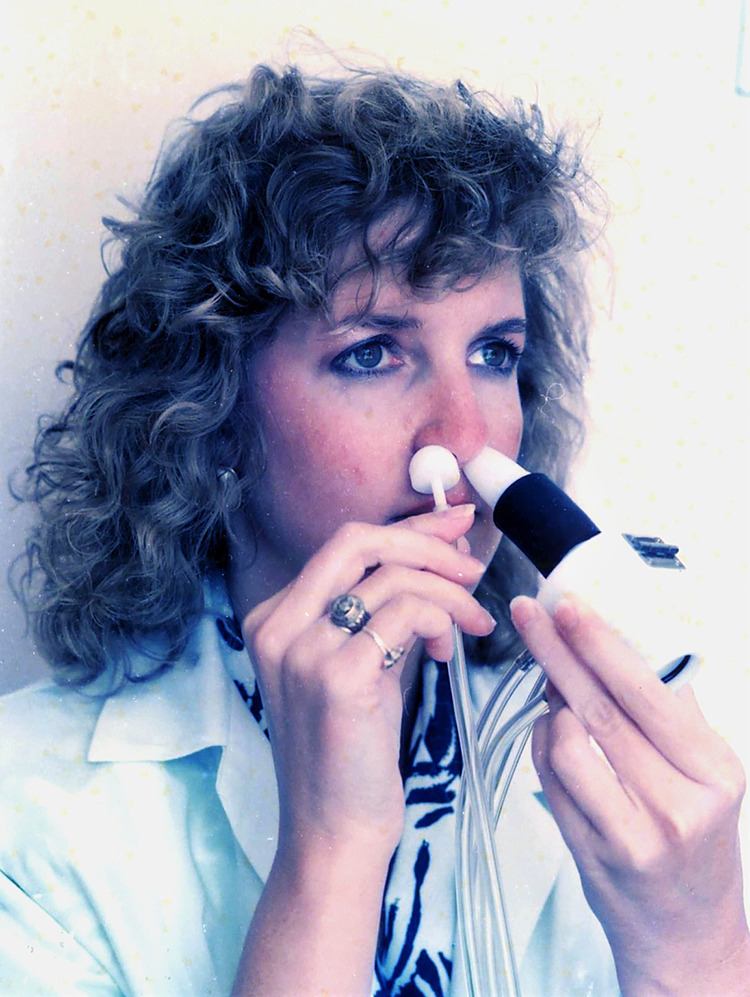
Active rhinomanometry with a nozzle.

**Figure 6:**
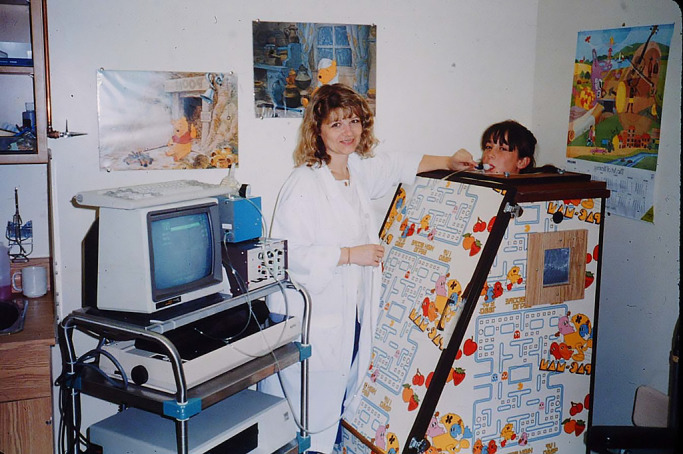
Head-out body plethysmograph (Nasal Airflow Laboratory, University of Toronto, Canada).

Active rhinomanometry requires a mask (Fig. 4), a nozzle (Fig. 5), or a body plethysmograph (Fig. 6) for measurement of nasal airflow. The body plethysmograph might be the predominant device because *R* measured by this instrument is not affected by masks or nozzles placed on the nose.^41^ However, a body plethysmograph is a large device that requires a large space, and many clinicians hesitate to use it. Miljeteig et al.^42^ found almost identical *R* values measured by a head-out body plethysmograph with or without a mask. Thus, ISCOANA has recommended active anterior rhinomanometry with a mask as a standard method.^3,4^ In contrast, we compared *R* values measured by a head-out body plethysmograph with and without a mask or a nozzle on the nostril.^43^ There was a significant difference between the two groups with and without a mask but no significant difference with and without a nozzle.^43^ JSCOANA also applied a nozzle instead of a mask for active anterior rhinomanometry as a standard technique in Japan.^6^

The inconsistencies in these measurements might be caused by anatomical differences among human races. We compared *R* values and nostril sizes and shapes in normal adult Whites, Asians, and Blacks and found significant differences among them.^34^ Further discussions are still required for better international agreement.

### 3.2. Expressions of rhinomanometry

Most researchers in nasal physiology and pathology simultaneously determine $\dot{V}$ and *ΔP* during spontaneous nasal breathing, and *R* can be calculated using Eq. 1. The equation describes the relationship between *ΔP* and $\dot{V}$ under laminar flow conditions. However, the nonlaminar condition prevails during much of a quiet nasal breathing cycle.^44^ This discrepancy between empiric measurement and the equation is still unresolved.

ISCOANA has recommended measuring *R* at *ΔP*=150 Pa,^2–4^ whereas JSCOANA has recommended measuring *R* at 100 Pa in Japanese adults.^6^ In practice, even in Whites, we found that 24% of 285 measurements did not reach the point of *ΔP*=100 Pa.^23^ To measure *R* at these points, voluntary hyperventilation is required instead of quiet breathing in many cases; however, voluntary hyperventilation is neither quiet nasal breathing nor physiologically normal.

Therefore, equations can be adjusted to fit a pressure–flow curve. Röhrer^45^ suggested the formula

(5) $$\Delta P=k_{1}\dot{V}+k_{2}{\dot{V}}^{2}\text{,}$$

where *k*_1_ is the coefficient of laminar flow and *k*_2_ is the coefficient of turbulent flow.

We compared *R* values at 150 Pa from Eq. 5 with actual measurements at the same point to determine the usefulness of the method.^38^ The relationship between calculated and measured *R* values at 150 Pa on expiration was

(6) MR=0.99CR+0.01,

and on inspiration was

(7) MR=0.93CR+0.02,

where MR is the measured resistance and CR is the calculated resistance from Eq. 5.

The *R* values from Eq. 5 were almost identical to the actual measurements on both expiration and inspiration. We considered the calculated *R* values at 150 Pa from Eq. 5 to be comparable with the actual measurements; additionally, we were able to estimate *R* at any predetermined points regardless of whether the pressure–flow curve attained the point (e.g., *Δ*P=75, 100, and 150 Pa).^23^

Fischer^46^ revealed that the pressure–flow curve for defining *R* is better described by

(8) $$P=\Delta P/{\dot{V}}^{n}$$

where *n* is the coefficient of nonlaminar flow.

Eichler and Lenz^16^ demonstrated *n* to be 1.85, and Dallimore and Eccles^47^ assumed *n*=2. They believed that most airflow through the nose during spontaneous breathing should be turbulent. In our further studies of Japanese normal adults,^23^ we presented the exact relationship and coefficient as

(9) $$R=0.78(\Delta P/{\dot{V}}^{1.33})\text{.}$$

Richerson and Seebohm^48^ demonstrated that *n*=1.5 for occidental normal adult individuals and found that the characteristics of airflow through the nose are transitional and not laminar or turbulent. However, these complex equations and coefficients are not used in the clinical setting.

Further international discussions are required to establish a proper standard, even for *R* in adults. The current Japanese and international standards for adults are shown in Table 1.^4,6^

**Figure 7:**
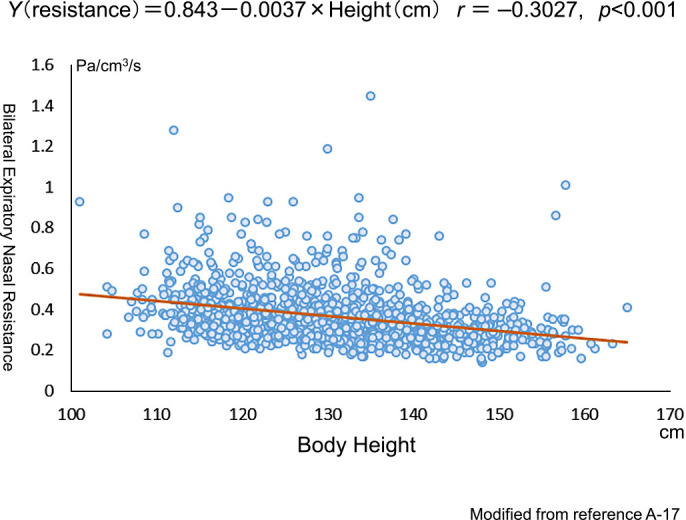
Relationship between expiratory nasal resistance and body height in Japanese healthy children. Modified from Ref. 50.

A single study on *R* values in children worldwide has been conducted.^49^ JSCOANA proposed the standard values of *R* in Japanese children in 2018 (Fig. 7).^50^

Many studies have been performed to investigate the structures that mainly generate resistance in a healthy nose.^51–57^ The anterior nostril, including the nasal valve; the anterior portion of the inferior nasal turbinates; and the natural anterior septal deviation of the nose are assumed to be the origins of resistance in a healthy nose. However, anatomical racial differences must be taken into account.^34^

### 3.3. Physiology of the nasal airway

In healthy individuals, *R* changes because of volume changes in the nasal mucosa under different conditions, such as the nasal cycle,^58–60^ body position,^61^ pressure on the lateral body,^62^ exercise,^47,48,63^ nasal breathing behavior,^47,64^ and ambient air conditions.^47,63,65–67^

According to Cole,^68^ even at rest, humans breathe 400 L/h of air at outdoor temperatures of –88°C to 58°C as measured at the South Pole and Libya, respectively. The most important functions of the nose are to warm and humidify inspired air during its passage through the nose.^68^ This air conditioning is of greatest importance for protection of the lower respiratory tract mucosa. The mucosa of the nasal turbinates plays a role in this air conditioning.^69,70^

The volume of the normal nasal mucosa is spontaneously altered because of a reflex induced by several types of stimuli, and the rhythm of autonomic nervous system activity is mainly regulated by mucosal blood flow.^69^ Studies have been performed to investigate local thermal and humidified stimulation of the human body, such as that on the skin of the dorsal thorax, foot, or hand.^66,67,70^ Solomon^70^ observed nasal obstruction instead of an increase in nasal patency during cold exposure. The mechanism of an immediate nasal response to local cooling is most likely a reflex-induced decrease in nasal blood flow, similar to the vasoconstrictor response in the skin.^70^

No studies have focused on the nasal airway under completely controlled environmental temperature and humidity. We measured *R* under totally controlled environmental temperature and humidity in atmospheric condition–controllable rooms in healthy individuals and patients with allergic rhinitis in the sitting position without exercise to prevent the effects of posture and exercise.^71^ Rapid changes in the ambient air temperature from warm (35°C) to cool (7°C) significantly increased *R* in patients with allergic rhinitis but not in healthy subjects, regardless of whether the air was dry or moist. Additionally, *R* in patients with perennial allergic rhinitis increased after heated (42°C) aerosol inhalation through the nose, whereas no changes occurred in healthy subjects with hyperthermal stimulation.^72,73^ An allergic nasal mucosa responds more strongly to various ambient or local irritants than does a healthy mucosa. We also observed markedly reduced *R*^61,74^ and more pronounced effects of exercise on the nasal mucosa in patients with allergic rhinitis than in healthy subjects.^75^

Autonomic alternative changes in nasal congestion and decongestion occur in healthy humans. The apparently spontaneous nasal cycle is irregular in frequency and amplitude, and reciprocity between sides is fairly constant. The term “nasal cycle” is well established and widely recognized.^76^ There is no significant change in the total resistance of the combined nasal cavities in a healthy nose. Although severe unilateral obstruction has been noted in both recumbent and sleeping subjects, an increase in resistance of the congested nasal cavity does not exert a major effect on total resistance.^77^ The nasal cycle and its modifications are sensitive indicators of widely distributed, reciprocating neuroautonomic activity, and although an interesting source of speculation, these vascular responses do not necessarily perform a functionally useful role in the nose.^44^

When evaluating the nasal airway, the physiological influence of the human body, including the nasal cavity locally, must be considered. In Japan,^6^ to prevent this possible influence, subjects must relax in the sitting position for 10 min before *R* measurement under ordinary room temperature and humidity (Table 1).

### 3.4. Relationship between subjective and objective nasal obstruction

Rhinomanometry is a useful clinical method of objectively evaluating nasal congestion worldwide. However, it is not unusual to find a patient in whom the subjective perception of nasal obstruction is inconsistent with the measured resistance to airflow.^64^ Such discrepancies can puzzle not only the clinician but also the patient.

Bachmann and Nieder^78^ compared *R* values with the sensations of nasal obstruction and rhinoscopic findings of 117 patients and found that all 3 parameters indicated good agreement in 75% of the patients. McCaffery and Kern^79^ studied 1000 patients and found that *R* was closely correlated with the severity of any obstructive symptoms, but they did not grade their rhinoscopic findings. We examined 101 Canadian patients and found that the *R* values were consistent with the rhinoscopic findings but not with the severity of chronic nasal obstruction.^80^ Similarly, the relationship between objective evidence of nasal patency and subjective nasal congestion is unresolved.

Eccles and Jones^81^ studied *R* and the sensation of nasal patency before and after 5 min of exposure to menthol vapor. The authors found no consistent effect on *R* despite the majority of subjects being aware of an increased nasal patency sensation. In addition, nasal anesthesia, including anesthesia of the vestibule, decreased the action of menthol vapor in enhancing the sensation of nasal airflow.^81^ Inhaling menthol vapor stimulates nasal cold receptors, causing the brain to misinterpret sensory information and leading to an increased sensation of nasal patency with no accompanying decrease in *R*.

Stimulation of the palate by L-menthol enhanced the nasal sensation of airflow without affecting objective nasal patency.^82^ In addition, the enhanced sensation significantly decreased by surface anesthesia of either the palatal or nasal mucosa.^83^ Direct stimulation of the sensory nerve endings of the palatal and nasal mucosa by L-menthol increases subjective nasal patency without an accompanying increase in objective nasal patency.

Burrow et al.^84^ reported that although exercise significantly decreases *R*, relatively few subjects noticed any cold sensation or increased awareness of nasal patency after exercise. Similarly, we compared *R* values with the degree of subjective sensation of nasal congestion before and after decongestion of the nasal mucosa by a topical decongestant and found appropriate agreement between objective and subjective nasal patency in only half of the subjects.^85^

The relationship between subjective and objective nasal patency in Canadian patients differs from that in Japanese patients.^24,27,86^ To determine why, we investigated the characteristics of the sensation of nasal congestion and *R* in both groups.^36^ The mean severity of the perception of nasal obstruction was significantly higher in Canadian patients than in Japanese patients, but there was no significant difference in *R*. In the Canadian patients, the reasons for nasal obstruction were directly related to nasal breathing (e.g., “requires an effort to breathe through the nose” and “no clear feeling after blowing the nose”), whereas in the Japanese patients, the reasons for nasal obstruction were related to indirect factors (e.g., “unable to concentrate on job or study” and “nasal speech pointed out by another person”). Therefore, these differences may be related to nationality and anthropological characteristics.

**Figure 8:**
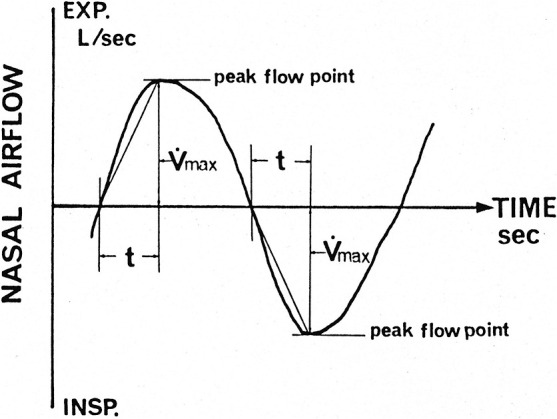
Acceleration of nasal airflow calculated as $A={\dot{V}}_{\max}/t$, where *A* is acceleration (L/s^2^), $V_{\max}$ is airflow at the peak flow point (L/s), and *t* is the time from commencement of breath to the peak flow point (s) on the nasal airflow curve. Modified from Ref. 86.

In considering possible aerodynamic indicators of subjective nasal congestion, the characteristics of nasal breathing behavior other than *R* might be important. To express the salient features of nasal breathing behavior, we measured the acceleration of nasal airflow^86^:

(10) $$A=\dot{V}\max/t\text{,}$$

where *A* is acceleration, ${\dot{V}}_{\max}$ is airflow at the peak flow point, and *t* is the time from the commencement of breath to the peak flow point (Fig. 8).

**Figure 9:**
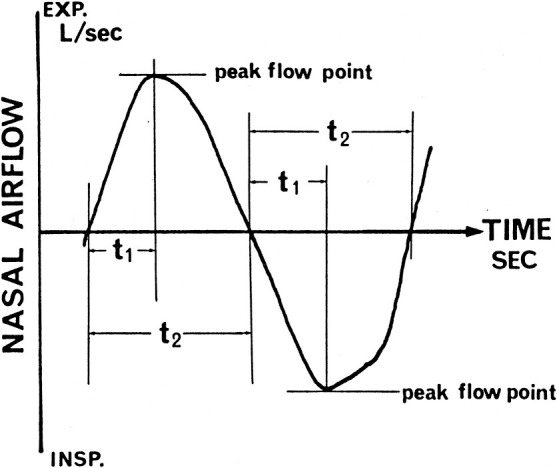
Peak flow index (PFI)=*t*1/*t*2, where *t*1 is the time from commencement of breath to the peak flow point and *t*2 is the duration of entire inspiration or expiration on the nasal airflow curve. Modified from Ref. 86.

The peak flow index (PFI) is then calculated as

(11) $$\mathrm{PFI}=t_{1}/t_{2}\text{,}$$

where *t*_1_ is the time from the commencement of breath to the peak flow point and *t*_2_ is the duration of the entire inspiration or expiration (Fig. 9).

During quiet nasal breathing, the nasal breathing behavior in subjects without congestion tended to exhibit higher acceleration and a smaller PFI on inspiration through a wide range of R values.^86^ The nasal airflow acceleration and the PFI are useful as objective indicators of the sensation of nasal congestion and can complement the diagnosis of subjective nasal obstruction.

The relationship between objective evidence of nasal patency and subjective symptoms is still unclear, and further studies are needed to define useful subjective criteria in patients with nasal obstruction.

### 3.5. Acoustic rhinometry

**Figure 10:**
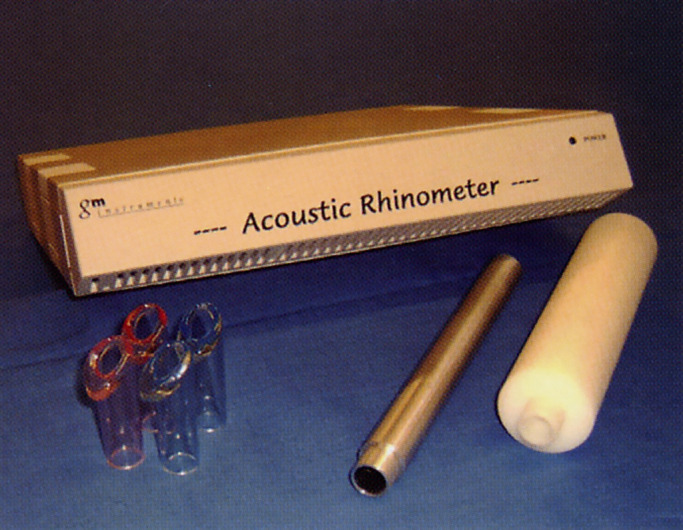
Acoustic rhinometer (GM Instruments, Irvine, Ayrshire, Scotland).

In 1989, acoustic rhinometry was developed as another method of objectively assessing nasal patency (Fig. 10).^37^ Acoustic rhinometry can assess the narrowest part in the nasal cavity without difficulty. The underlying principle^87,88^ is as follows. An audible sound (150–10,000 Hz) propagating in a tube is reflected by local changes in acoustic impedance. Assuming no losses and no changes in acoustic impedance, the sound travels forever in the tube. In a tube with an alternating diameter or in the nasal cavity, the sound is reflected by changes in acoustic impedance due to the alternating cross-sectional area. If the signals of the incident and reflected waves are recorded in a time domain, we can determine the distance to the local impedance change; additionally, by comparing the incident and reflected waves, we can determine the size of the change in the cross-sectional area. We can then measure consecutive cross-sectional areas and volumes in the nose using acoustic rhinometry.

In 2004, Saito et al.^89^ were the first to determine the actual sound direction in the nasal cavity using an acoustic rhinometer and the exact cross-sectional area angles in the nasal cavity using an adult-size artificial nasal model scanned by three-dimensional computed tomography and acoustic rhinometry. The most identical values of the cross-sectional areas between those measured by computed tomography and by acoustic rhinometry were the combined line between the top and bottom of the nasal cavity equivalently deferred from the anterior nostril. Consequently, the most feasible direction of sound waves in the nasal cavity was the sequence of 90° to these cross-sectional area inclinations. The cross-sectional areas (cm^2^) at the I-notch (isthmus nasi) and C-notch (head of the inferior turbinate) (both points are the so-called minimum cross-sectional area of the nose) and volumes (cm^3^) from the nostril to the posterior 4 cm (0–4 cm) and the posterior 7 cm (0–7 cm) are representative evaluating expressions of acoustic rhinometric measurements. Acoustic rhinometric measurements only demonstrate width; they do not explain the shapes of the nasal cavity or the aerodynamic physiology.^90^ We feel that rhinomanometry and acoustic rhinometric measurements are valuable as objective indicators of the nasal airway, but we should consider the characteristics of the two methods.

Lane et al.^88^ demonstrated that the minimum cross-sectional area was not correlated with the sensation of nasal obstruction in patients with hay fever, while Roithman et al.^91^ found a significant relationship between the unilateral minimum cross-sectional area and the sensation of nasal obstruction. However, they found no simple relationships between rhinomanometry and acoustic rhinometry.^91^ This is why the two methods probably do not reveal simple mathematical relationships.^88^ We compared the perception of nasal obstruction by rhinomanometric and acoustic rhinometric assessments before and after nasal or sinus surgery in 50 Japanese patients.^90^ Before surgery, there were significant relationships between the *R* values on expiration or inspiration and the perception of nasal obstruction and nasal volume (0–4 and 0–7 cm). Subjective nasal congestion, *R*, and acoustic rhinometric expression (except for the cross-sectional areas at the I-notch) significantly improved after surgery. Acoustic rhinometry facilitates static and anatomic evaluation of the nasal cavity, while rhinomanometry facilitates dynamic and physiological assessment. However, these 3 factors of nasal patency relatively coincided with each other in the 50 Japanese patients. The relationship between objective and subjective nasal patency in patients from different countries may be associated with nationality and anthropological characteristics. These factors make mutual relationships more difficult to identify.

ISCOANA has still not established international standards for how to evaluate and average acoustic rhinometric values. JSCOANA is currently trying to finalize standard values for normal Japanese adults. After finalization, we can discuss the differences in the standards of acoustic rhinometry between Japan and other countries.

### 3.6. Allergic rhinitis and nasal obstruction

In recent decades, the number of individuals in Japan with specific immunoglobulin E to mites or pollen has significantly increased.^92^ Many Japanese people have nasal obstruction due to allergic rhinitis. Bilateral nasal resistance (*T*) due to cedar pollen in patients with pollinosis moderately coincides with floating pollen counts.^93–96^ Furthermore, in patients with hay fever, OSA is aggravated during the pollinating season.^97^ Patients with allergic rhinitis are treated with oral or topical medicines or surgery. Determining the grades and sites of nasal obstruction may be important for accurately diagnosing and treating allergic rhinitis. How do we, as clinicians, accurately evaluate the grades and sites of nasal obstruction in patients with allergic rhinitis? How do we objectively evaluate the effectiveness of treatment? Can exposure of the nasal mucosa to antigen or histamine in patients with allergic rhinitis help to objectively evaluate the severity? Several investigators have compared the subjective sensation of nasal congestion using a visual analog scale (VAS), intranasal inspection, and objective assessments of nasal obstruction by rhinomanometry or acoustic rhinometry before and after exposure of the nasal mucosa to antigen or histamine.^75,98,99^ The recent European Academy of Allergy and Clinical Immunology (EAACI) position paper on nasal antigen provocation tests in patients with allergic rhinitis reported how to evaluate perceptive nasal obstruction, rhinoscopic findings, and objective measurements by rhinomanometry or acoustic rhinometry.^100^ We can use this position paper for suitable evaluation of patients with allergic rhinitis in daily practice.

Active anterior rhinomanometry, the standard method of objectively assessing nasal patency recommended by ISCOANA and JSCOANA, cannot measure *R* in patients with complete nasal blockage after antigen exposure. This is a disadvantage of active anterior rhinomanometry in antigen challenge tests. Therefore, we believe that acoustic rhinometry is preferable in antigen or histamine challenge tests of the nasal mucosa because few patients are excluded after stimulation.^101^ Wakabayashi et al.^102^ used acoustic rhinometry to evaluate the effectiveness of pranlukast dry syrup in children with cedar pollinosis before and after pollen exposure.

Using intranasal heated aerosol inhalation to cause local hyperthermia is an effective and safe treatment, especially for pregnant women with allergic rhinitis. Determining the underlying local hyperthermia in patients with allergic rhinitis is important.^86,103^ We compared nasal *R* values in two groups of patients with perennial allergic rhinitis: those treated with hyperthermia and those who were untreated. We found that the *R* values after a house dust challenge to the nasal mucosa were significantly lower in the hyperthermia group than in the untreated group.^72^ Furthermore, in another study, the *R* values after a histamine challenge showed no significant differences between the treatment group and healthy subjects.^73^ Local heated aerosol inhalation therapy may suppress both antigen-specific and antigen-nonspecific hypersensitivity of the nasal mucosa in patients with allergic rhinitis.

### 3.7. Nasal obstruction and sleep disorder

Nasal obstruction may accompany OSA. In 1992, Miljeteig et al.^104^ found no significant differences in *R* values measured by active anterior rhinomanometry between patients with OSA and healthy subjects. However, some investigators doubt the conclusion of this single report and emphasize that nasal obstruction can cause impaired quality of sleep and contribute to OSA.^105–107^

Li et al.^108^ compared high- and low-*R* groups of patients with symptoms suggestive of OSA and found that the high-*R* group had a significantly higher snoring index and that a unilateral high *R* was significantly correlated with the respiratory disturbance index, which is an indicator similar to the apnea–hypopnea index. Zwillich et al.^109^ showed that apneas, sleep arousals and awakenings, and loss of deep sleep occur during nasal obstruction. Friedman et al.^110^ reported that most patients with OSA are aware of improvement in nasal and sleep symptoms after correction of nasal airway obstruction, but nasal surgery alone does not consistently improve OSA. They also noticed that nasal airway reconstruction may contribute to a decrease in continuous positive airway pressure levels and improvement in oxygen saturation. Nakata et al.^111^ showed that nasal surgery helps to improve sleep quality and daytime sleepiness.

In their study of 911 participants, Young et al.^112^ showed that patients with rhinitis are significantly more likely to report habitual snoring or chronic excessive daytime sleepiness and that habitual snorers have significantly lower nasal airflow than nonsnorers. Colas et al.^113^ found that sleep quality is worse in patients with severe allergic rhinitis and that nasal obstruction is associated with poorer sleep quality. Hara et al.^114^ discovered that 4 weeks of pranlukast administration in patients with perennial allergic rhinitis improves sleep disorder symptoms and is significantly correlated with improvement in nasal congestion.

Overall, nasal obstruction is significantly associated with not only snoring, daytime somnolence, and sleep quality but also OSA. Rhinologists must consider the possibility that nasal obstruction can accompany sleep disorders.

Kihara^115^ found that the rhinomanometric and acoustic rhinometric results in children with OSA significantly improved after adenoidectomy or tonsillectomy for OSA treatment. Satisfactory sleep after surgery may regulate their physical conditions and secondarily increase their nasal patency.

### 3.8. Nasal resistance and headache

In Europe and North America, several reports have discussed chronic headache and facial pain due to chronic rhinosinusitis that was treated by surgery.^116–118^ In Japan, no such research has been performed. We studied 150 Japanese patients with chronic rhinosinusitis and found that 60% of the patients had headache or facial pain; additionally, rhinoscopic inspections, rhinomanometric results, and sinus X-rays revealed no consistent relationships with the presence, site, or severity of craniofacial pain.^119^ Neurotic factors might play a significant role in causing headache and facial pain. Therefore, we used the Cornell Medical Index-Health Questionnaire (CMI)^120^ to assess neurotic tendency in the following series.^121^ Although no significant differences in CMI grading were found between the headache and pain-free groups, individual variability could not be excluded. In the same study, 85% of the patients noted cure or improvement of headache or facial pain after surgery. However, no significant differences were found in the postoperative reduction rate in *R* values between the headache and pain-free groups, indicating that increased *R* alone is not sufficient to cause pain in most patients. Further investigations are still needed to determine the cause of this pain.

### 3.9. Applying rhinomanometry to objective evaluation of hypernasality

**Figure 11:**
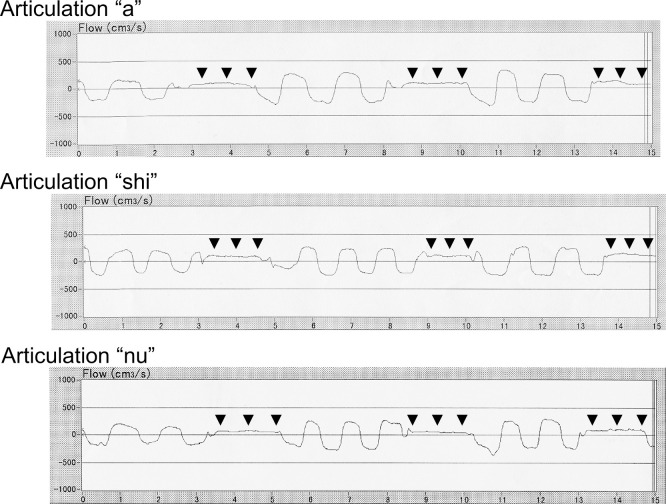
Aerodynamic assessment reveals velopharyngeal incompetence. Airflow leakage through the nose was observed during articulation of “a” and “shi” (arrowheads). Even in healthy and competent children, airflow can normally be detected during articulation of “nu” (arrowheads).

**Figure 12:**
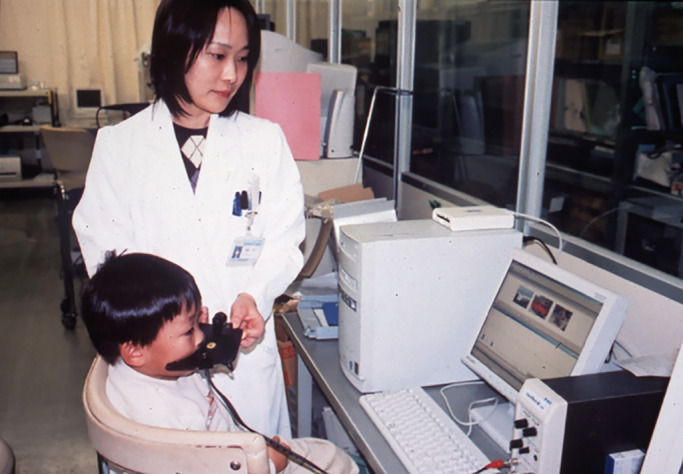
Nasometer II 6450 (KayPENTAX Inc., Lincoln Park, NJ, USA).

Hypernasality caused by velopharyngeal incompetence is a major cause of articulation disorders in children with cleft palate.^122,123^ After palatal closure surgery in these children, speech therapy is required to achieve proper articulation. However, hypernasality remains in some cases despite the appropriate performance of postoperative speech therapy,^124^ and pharyngeal flap construction surgery is therefore required. However, attention must be paid to complications such as mouth breathing, snoring, OSA, underdevelopment, otitis media with effusion, bradycardia, and cardiac arrest after these surgical procedures.^124^ The indication for pharyngeal flap construction surgery should be strict and impartial. To appropriately evaluate velopharyngeal incompetence in these children, rhinomanometry is used to detect airflow leakage through the nose during articulation of “a,” “shi,” and “nu”.^124^ We detected airflow leakage through the nose during articulation of “a” or “shi” in children with velopharyngeal incompetence and during articulation of “nu” in healthy children, and competent and incompetent children (Fig. 11). This objective assessment is easy to perform in children because of its painlessness and simplicity. We measured nasal airflow during articulation in 3-year-old children with cleft palate in the same way as we measured *R* in healthy children of the same age. Recently, for more exact evaluation of velopharyngeal incompetence, a nasometer (KayPENTAX Inc., USA) (Fig. 12) has been used.^124^ These systems of objective assessment of velopharyngeal incompetence have been set up in our laboratory in Japan, and many children with cleft palate have been sent to our hospital from all over the country. Pernasal fine and flexible fiberscopy, lateral facial X-rays, and acoustic impressions by skilled speech therapists are primarily important for direct inspection of the soft palate through the mouth. In addition, the patients’ intelligence and family circumstances must also be comprehensively evaluated to strictly decide whether pharyngeal flap construction surgery is required. We believe that aerodynamic measurements of nasal airflow during articulation in children with cleft palate could be effective.

## 4. Conclusion

Objective assessment of nasal patency is well established, but several minor problems remain to be resolved (e.g., methodologies, equipment, expressions, and racial differences). Thus, continuous research is required to develop more appropriate and simplified methods and equipment for objective assessment of nasal patency. Furthermore, this superior procedure of objective assessment of nasal passage should be expanded into the domain of the relationship between nasal breathing and sports because exercise and nasal or mouth breathing are important in the field of sports medicine.^74,125^ Nasal patency or obstruction is pathophysiologically important. We hope this review is regarded as the current landmark of research on the objective assessment of nasal obstruction.

## Figures and Tables

**Table1:** Characteristics of the Japanese and international standards of rhinomanometry

	Japanese	International
Method	active anterior with nozzle or mask	active anterior with mask
Expression	nasal resistance at *Δ*100 Pa	nasal resistance at *Δ*150 Pa
Unit	Pa/cm^3^/sec	Pa/cm^3^/sec
Equipment	authorized by JIS (Japanese Industrial Standard)	undecided
Examinee position	in sitting position	in sitting position
Resting duration before exam.	10 min	undecided
Breathing manner	spontaneous breathing	spontaneous breathing
Normal value of adult	0.25±0.12 Pa/cm^3^/sec	undecided
